# Kinematic instrumental assessment quantifies compensatory strategies in post-stroke patients

**DOI:** 10.1007/s11517-025-03439-2

**Published:** 2025-09-15

**Authors:** Alessandro Scano, Eleonora Guanziroli, Cristina Brambilla, Alessandro Specchia, Lorenzo Molinari Tosatti, Franco Molteni

**Affiliations:** 1 Institute of Intelligent Industrial Technologies and Systems for Advanced Manufacturing, Laboratory of Advanced Methods for Biomedical Signal and Image Processing, Italian National Research Council, Milan, Italy; 2 Villa Beretta Rehabilitation Center, Ospedale Valduce, Costa Masnaga, Italy

**Keywords:** Kinematics, Upper-limb, Compensatory movements, Post-stroke, Hemiplegic, Fugl-Meyer Assessment, Kinect

## Abstract

In clinical practice, the upper limb function of hemiplegic post-stroke patients is commonly evaluated using clinical tests and questionnaires. Performing a reliable investigation of compensatory strategies adopted for the upper limb movement may shed light on the basis of motor control and the mechanism of functional recovery. To quantitatively evaluate the compensatory strategies in post-stroke hemiplegic patients, we conducted an observational study in which 36 hemiplegic patients were enrolled and were stratified according to the Fugl-Meyer score. We assessed compensatory strategies in upper limb movements, specifically reaching (RCH) and hand-to-mouth (HTM) movements, using the Kinect V2 device. 11 severe, 8 severe-moderate, 9 moderate-mild, and 8 mild patients and 17 controls participated in the study. Our results showed that severe, severe-moderate, and moderate-mild patients can be discriminated from healthy participants in almost all parameters. In particular, patients showed a reduced ROM of the shoulder in RCH, higher shoulder and elbow vertical displacement, and lower wrist vertical displacement in HTM. Interestingly, compensatory parameters also discriminate mild patients from healthy controls, such as head frontal and vertical displacements. Our protocol works effectively and the instrumental assessment of compensatory strategies in post-stroke patients allows to discriminate different levels of impairments even with low-cost devices.

**Graphical Abstract:**

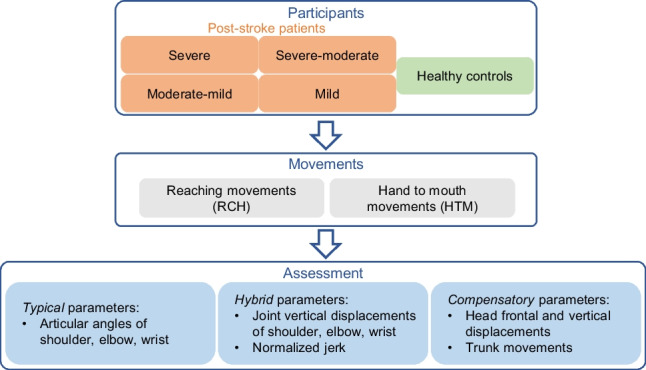

## Introduction

Stroke is one of the major leading causes of death worldwide and it is the third cause of disability in adults: it is estimated that stroke affects more than 12 million new people every year [1]. Post-stroke patients usually suffer from motor impairments of the upper limb, that may limit their independence in activities of daily living. Movement deficits are more evident in the contralesional side and are characterized by muscle weakness, abnormal muscle tone, and loss of inter-joint coordination [2]. Patients compensate for these deficits with available motor strategies, such as movements of the trunk and scapula that allow to accomplish motor tasks [3] by exploiting the redundancy of the musculoskeletal system [4]. In some cases, compensation strategies can be mistaken for motor recovery, since some of these strategies may not be detected by low-sensitive behavioral measures [5, 6].

Thus, in order to quantify and improve motor recovery, it is fundamental to correctly evaluate the motor impairment of patients and to identify compensatory strategies. Clinicians usually administer assessment scales, such as the Fugl-Meyer Assessment (FM) [7] or the Wolf Motor Function Test (WMFT) [8], for motor function evaluation. However, compensatory strategies, such as displacements of the trunk in reaching movements, are not explicitly measured by most activity-level motor scales, with the exception of the WMFT [9, 10]. Therefore, quantitative assessments, such as the evaluation of kinematic and dynamic parameters, may allow more complete and objective measures. Compensatory strategies were previously analyzed with kinematic and kinetic measures with marker-based motion capture systems [11, 12]. However, these technologies cannot be used in small clinics and at home. Furthermore, rehabilitation is usually delivered by physiotherapists in clinics, leading to the need of staff and structures. The increase in the aging population that needs clinical assistance and rehabilitation has stressed the lack of medical staff and structures and the high costs related to rehabilitation monitoring [13]. Moreover, the COVID-19 pandemic crisis emphasized the necessity to have devices for telerehabilitation and home monitoring [14]. In this scenario, cost-effective and portable solutions are valuable. They guarantee patient monitoring and evaluation at home or in small centers [15]. Assessments and therapies delivered at home allow to reduce costs and improve the accessibility and efficiency of rehabilitation and to guarantee the continuity of care [16].

Kinect sensors are a valuable tool for patient monitoring and allow kinematic evaluations as they embed an SDK for human skeleton tracking. Kinect-based rehabilitation offers a low-cost and flexible method, providing rehabilitation exercises based on video games [17] that promote motivation and adherence to the treatment to improve patients’ motor performance [18]. The Kinect sensor allows interaction with the game without using wearable devices and without the need of physiotherapists [19]. A recent review highlighted that combining Kinect-based rehabilitation therapy with standard physiotherapy has positive effects on performance improvements in stroke patients [20]. However, most of the studies did not use biomechanical Kinect-based assessments for the evaluation of motor performance, but they relied on clinical scales. Original protocols for biomechanical assessments based on the Kinect technology were developed in a few studies. These allowed to distinguish the performance of post-stroke patients with respect to healthy participants in reaching movements [21]. The reliability of markerless Kinect V2 was assessed in multiple functional movements, proving to be able to discriminate patients from healthy participants [22]. Among others, the Kinect sensors were used for evaluating walking tests in patients with multiple sclerosis [23] and for measuring clinically relevant movements in patients with Parkinson’s disease [24]. Moreover, associating Kinect data with machine learning techniques, previous studies accurately classified postures of stroke patients [25]and also provided corresponding WMFT-FAS scores [26]. However, few studies so far have investigated and quantified how patients adopt compensatory strategies to produce motion, especially when using markerless devices. Compensatory movements, especially regarding trunk movements, were usually assessed with marker-based tracking systems. In a preliminary study, post-stroke patients with moderate impairments showed higher trunk flexion during reaching movements with respect to healthy controls [27]. Abnormal trunk flexion was investigated in other studies on post-stroke patients with different levels of impairment [9, 28]. In particular, in post-stroke patients with different levels of disability (from severe to mild), both forward trunk displacement and trunk torsion were recognized as compensatory strategies when reaching a target [2]. Moreover, another study found correlations between the increase of shoulder motion and a decrease in trunk displacements during rehabilitation in moderate post-stroke patients [29]. Two studies investigated compensatory strategies with the Kinect in mild and moderate post-stroke patients during reaching movements, showing an abnormal frontal and lateral displacement of the trunk with respect to healthy controls [21, 30]. Since markerless devices demonstrated their feasibility in biomechanical assessments, they could also be used for investigating the presence of compensatory strategies, providing a more precise kinematic assessment of the patients.

Following the need for standardized tools for assessing motion and compensatory strategies in post-stroke patients, in this study, we propose a protocol based on functional movements, already used in clinics, that allows motor assessment of adults and post-stroke patients with the use of Kinect technology. The protocol consists of a kinematic assessment, including motor control parameters. The parameters of the assessment were categorized in (1) typical of the movement, i.e., specific for the correct movement execution; (2) compensatory, i.e., degrees of freedom or movements that should not be involved in the execution of the movement but they are used by patients to compensate the motor impairment; and (3) hybrid parameters (partially compensatory), i.e., parameters that are involved in the movement execution but that can be recruited abnormally by patients as a part of a compensatory strategy. In order to test the usability of the proposed instrumental protocol, we evaluated 36 hemiplegic patients, divided into subgroups based on the severity of impairment of the contralesional limb (as measured by FM-UL) (reference subgroups), and we compared them to 17 age-matched control volunteers. Our protocol works effectively with low-cost devices, and it can distinguish not only patients from healthy participants but also patients with different severities of impairment.

## Methods

### Recruitment

We conducted an observational study in which kinematic data of the contralesional limb of stroke patients were compared to age-matched controls performing reaching movements (RCH) and hand-to-mouth (HTM) movements [21, 31, 32]. Both RCH and HTM are multi-joint movements requiring multi-joint coordination. Patients were also evaluated with the Fugl-Meyer Assessment scale in order to identify the severity of their impairment.

Two cohorts of participants were included in this study. Eligible participants were post-ischemic or hemorrhagic stroke patients, with unilateral upper limb deficit, recruited by the Villa Beretta Rehabilitation Center, Ospedale Valduce (Costa Masnaga, Italy) within the ethical approval granted by the section “IRCCS Fondazione Don Carlo Gnocchi”, Comitato Etico IRCCS Regione Lombardia (study ID 03_08/02/2023). The experimental trial was conducted in compliance with the Declaration of Helsinki. Inclusion criteria included: ischemic/hemorrhagic stroke survivors; unilateral upper limb deficit; ability to understand the instructions and ability to remain in a sitting posture. Exclusion criteria were as follows: bilateral impairment; cognitive impairment; and other severe medical problems. Healthy control participants were included if they were age-matched and without neurological or musculoskeletal impairments. Before testing, all healthy subjects were questioned and clinically evaluated for the presence of neurological or orthopedic signs, and excluded if any.

All healthy people and patients have given their informed consent for participation in the research study. Both healthy subjects and patients were then instrumented by an experienced bioengineer with a Kinect V2-based setup developed at the Italian Council of National Research (CNR).

### Data collection

Healthy people and patients followed a protocol presented in recent studies [31, 32]. The subjects sat on a chair, adjustable for height, with the feet resting on the floor and the knees and hips bent at 90 degrees. In the rest position, both hands were lying on the thighs, and the arms were positioned with flexed elbow and slightly extended shoulder. Starting from the rest position, subjects were asked to carry out the movements without moving their back away from the backrest. To perform the RCH movements, subjects had to move their hand toward a target located in front of them. In HTM movements, subjects had to move the hand toward the mouth. Tracking data were collected with in-house software for data recording coupled with Kinect SDK version 2.0. For both patients and controls, measurements were acquired in one session.

Patients were evaluated with the FM scale and they were divided into four subgroups based on the severity of their impairment, based on the cluster analysis results of Woytowicz et al. [33]: severely impaired patients with FM score ≤15, severe-moderate impaired patients with FM score > 15 and < 35, moderate-mild impaired patients with FM score ≥35 and < 54, and mild impaired patients with FM score ≥54.

### Data analysis

The evaluation protocol consistently extended the one presented in previous work [21]. All data analysis and pre-elaborations were performed offline in MATLAB (Mathworks, Natick, MA, USA).

In the pre-processing stage, all marker coordinates were filtered with a 3rd order low-pass Butterworth filter with a cut-off frequency of 6 Hz, in order to remove noise. Each trial was segmented into phases. Each phase started from the resting position and ended when the arm reached the target in RCH movements and when the hand reached the mouth in HTM movements. The starting and ending points were identified with a threshold algorithm applied to the velocity profiles of the shoulder flexion in the sagittal plane in RCH movements and of the elbow flexion in HTM movements, setting the threshold at 5% of the maximum value of the velocity.

The assessment parameters were subdivided into three categories: *typical* parameters, that are necessary for the execution of the movement and are expected to be well represented and referenced in healthy participants’ motion, while they might be altered in patients; *hybrid* parameters, which are related to the movement execution but they could be exaggerated/reduced in patients due to compensatory strategies; *compensatory* parameters, that are degrees of freedom or movements related to compensatory strategies, that should not be recruited (or very limitedly recruited) in healthy participants.

Typical parameters were computed as in [21] and include:

- Shoulder flexion angle (SF, in °);
- Shoulder adduction angle (SA, in °);
- Elbow flexion angle (EF, in °);
- Wrist flexion angle (WF, in °).

Hybrid parameters were:

- Shoulder vertical displacement (SV, in m);
- Elbow vertical displacement (EV, in m);
- Wrist vertical displacement (WV, in m);
- Normalized jerk (NJ) [34], as a measure of the smoothness of the movement.

Compensatory parameters were:

- Head forward/backward displacement (HF, sagittal plane, in m);
- Head vertical displacement (HV, vertical plane in m);
- Trunk torsion (TT, coronal plane, in °);
- Trunk forward/backward tilt (TF, sagittal plane, in °);
- Trunk lateral tilt (TL, frontal plane, in °).

Angular displacements were computed as follows:

$$SF=\mathrm{acos}\left(-{\overrightarrow{u}}_{y}\bullet \overrightarrow{SE}\right)$$

$$SA=\mathrm{acos}\left({\overrightarrow{u}}_{z}\bullet \overrightarrow{SE}\right)$$

$$EF=\mathrm{acos}\left(\overrightarrow{SE}\bullet \overrightarrow{EW}\right)$$

$$WF=\mathrm{acos}\left(\overrightarrow{EW}\bullet \overrightarrow{SH}\right)$$

$$TT=\mathrm{acos}\left({\overrightarrow{u}}_{z}\bullet \overrightarrow{TR}\right)$$

$$TF=\mathrm{acos}\left({\overrightarrow u}_y\bullet\overrightarrow{TR}\right)in\;the\;sagittal\;plane$$

$$TL=\mathrm{acos}\left({\overrightarrow u}_y\bullet\overrightarrow{TR}\right)in\;the\;frontal\;plane$$

Denoting: *x* = *axis pointing forwards; y* = *vertical axis pointing upwards; z* = *horizontal axis pointing on the right of the participant; SE* = *shoulder-elbow unit vector; EW* = *elbow-wrist unit vector; WH* = *wrist-hand unit vector; u*_*y*_ = *vertical unit vector pointing upward; u*_*z*_ = *horizontal unit vector pointing laterally; TR* = *unit vector of the trunk, connecting two joints on the spine; SH* = *unit vector connecting right and left shoulder.*

Participants were positioned such that the sagittal axis corresponded to the x axis.

Joint displacements were computed as the distance between the position of the joint at the beginning and at the end of the movement, and the distance was normalized by limb length in order to compare different participants. Vertical displacements of the shoulder, elbow, wrist, and head were computed as the distance of each corresponding joint in the vertical axis y; the forward displacements of the head were computed on the sagittal axis x.

The range of motion (ROM) of each parameter was considered for analysis, defined as the difference between the maximum value and the minimum value during the movement.

### Statistical analysis

To determine the number of subjects to be enrolled, we used the software GPower 3.1.9.7 (Heinrich Heine University, Dusseldorf, Germany). The GPower a-priori test was performed on the assessment of shoulder flexion, estimating the effect size based on expected experimental data. When comparing healthy participants with the severe group, mean expected values for SF were 85° ± 10° for healthy participants and 40° ± 20° for patients, obtaining a very large effect size (> 2) and requiring only 5 subjects per group. When comparing healthy with the moderate group, the mean expected values of SF were 85° ± 10° for healthy participants and 60° ± 18° for patients, obtaining a large effect size (> 1) and requiring 8 subjects per group. The expected values for high-functioning patients (FM≥54) were almost indistinguishable from the expected values of healthy participants, leading to a very low effect size (0.13) requiring at least 640 subjects per group, which was beyond the possibility for this work. As a reasonable compromise, we recruited at least 8 participants per group.

All data distributions were tested for normality through the Kolmogorov-Smirnov test. For all the proposed variables of the assessment, a two-sample t-test was computed to compare pairs of subgroups. All pairs of subgroups were tested with a two-sample *t*-test (total number of tests for each parameter = 10). The significance level was set at 0.05, and Bonferroni corrections were used to limit the chance of type I error per comparison.

## Results

### Patients and controls’ characteristics

A total of thirty-six patients were enrolled in this study, and they were divided based on their FM score. The FM of each group was statistically different from the FM of the other groups (*p* < 0.001). 11 patients were in the severe subgroup (7F, 4M, 55.9 (13.3) years; FM = 7.91 (4.30)); 8 in the severe-moderate subgroup (4F, 4M, 50.7 (13.4) years; FM = 26.38 (5.50)); 9 in the moderate-mild subgroup (4F, 5M, 61.9 (17.2) years; FM = 40.00 (5.10)); 8 in the mild subgroup (2F, 6M, 63.1 (16.8) years; FM = 62.25 (3.58)). Seventeen age-matched control subjects were also enrolled (9F, 8M, age 55.6 (8.5) years).

### Evaluation outcomes

**Fig. 1:**
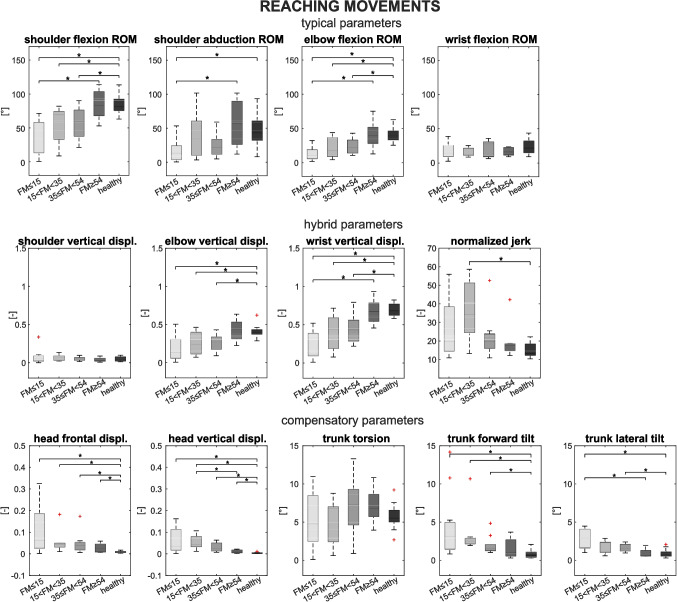
Parameters of the assessment for the RCH movement: in the first row, the distributions of typical parameters (SF, SA, EF, WF) are reported. In the second row, the distributions of hybrid parameters (SV, EV, WV, NJ) are reported. In the last row, the distributions of compensatory parameters (HF, HV, TT, TF, TL) are reported. Patient subgroups are shown in gray shades, and healthy participants are shown in black

A summary of the results for reaching movements is shown in Fig. 1 and statistical comparisons are summarized in Table 1.

**Table 1:** Mean inter-subject values of the parameters of the assessment for the RCH movement. Only significant *p*-values resulting from tests performed for all pairs between groups are reported

	FM≤15	15 < FM < 35	35≤FM < 54	FM≥54	Healthy	*p*
SF [°]	32.2 (25.2)	55.1 (27.6)	57.3 (26.0)	84.5 (22.0)	84.3 (14.2)	FM≤15 vs. h: *p* < 0.001 15 < FM < 35 vs. h: *p* = 0.003 35≤FM < 54 vs. h: *p* = 0.002 FM≤15 vs. FM≥54: *p* < 0.001
SA [°]	16.3 (15.7)	38.4 (35.5)	25.3 (19.6)	57.2 (35.2)	46.2 (23.9)	FM≤15 vs. h: *p* = 0.001 FM≤15 vs. FM≥54: *p* = 0.003
EF [°]	13.4 (9.8)	22.9 (15.9)	23.8 (11.5)	41.0 (19.7)	41.3 (10.8)	FM≤15 vs. h: *p* < 0.001 15 < FM < 35 vs. h: *p* = 0.003 35≤FM < 54 vs. h: *p* < 0.001 FM≤15 vs. FM≥54: *p* < 0.001
WF [°]	16.8 (11.1)	15.6 (6.5)	22.7 (11.2)	16.6 (5.7)	23.5 (10.3)	
SV [-]	0.08 (0.09)	0.06 (0.04)	0.06 (0.03)	0.04 (0.03)	0.05 (0.03)	
EV [-]	0.19 (0.18)	0.26 (0.16)	0.26 (0.11)	0.43 (0.14)	0.41 (0.07)	FM≤15 vs. h: *p* < 0.001 15 < FM < 35 vs. h: *p* = 0.003 35≤FM < 54 vs. h: *p* < 0.001
WV [-]	0.22 (0.18)	0.40 (0.24)	0.44 (0.18)	0.67 (0.16)	0.70 (0.08)	FM≤15 vs. h: *p* < 0.001 15 < FM < 35 vs. h: *p* < 0.001 35≤FM < 54 vs. h: *p* < 0.001 FM≤15 vs. FM≥54: *p* < 0.001
NJ [-]	27.6 (16.1)	36.9 (16.5)	22.6 (12.1)	19.5 (9.5)	15.1 (3.7)	15 < FM < 35 vs. h: *p* < 0.001
HF [-]	0.12 (0.12)	0.06 (0.06)	0.05 (0.05)	0.03 (0.02)	0.01 (0.004)	FM≤15 vs. h: *p* = 0.001 15 < FM < 35 vs. h: *p* = 0.002 35≤FM < 54 vs. h: *p* = 0.004 FM≥54 vs. h: *p* = 0.001
HV [-]	0.06 (0.06)	0.05 (0.03)	0.03 (0.02)	0.01 (0.01)	0.004 (0.003)	FM≤15 vs. h: *p* < 0.001 15 < FM < 35 vs. h: *p* < 0.001 35≤FM < 54 vs. h: *p* < 0.001 FM≥54 vs. h: *p* = 0.003 15 < FM < 35 vs. FM≥54: *p* = 0.004
TT [°]	5.14 (3.61)	5.09 (3.04)	7.24 (3.80)	7.14 (2.25)	5.73 (1.53)	
TF [°]	4.35 (4.30)	3.60 (3.13)	1.96 (1.26)	1.57 (1.36)	0.80 (0.54)	FM≤15 vs. h: *p* = 0.003 15 < FM < 35 vs. h: *p* = 0.002 35≤FM < 54 vs. h: *p* = 0.004
TL [°]	2.87 (1.25)	1.59 (0.86)	1.65 (0.51)	1.00 (0.49)	0.92 (0.47)	FM≤15 vs. h: *p* < 0.001 35≤FM < 54 vs. h: *p* = 0.001 FM≤15 vs. FM≥54: *p* < 0.001

#### Typical parameters

Healthy participants showed a higher ROM for typical parameters (especially for shoulder flexion and elbow flexion), while the severe group of patients showed a reduced ROM of articular angles. In particular, the SF of the healthy group was higher than severe (*p* < 0.001), severe-moderate (*p* = 0.003), and moderate-mild (*p* = 0.002), and the SF of the mild patients was higher than severe patients (*p* < 0.001). The SA of severe patients was lower than healthy (*p* = 0.001) and mild patients (*p* = 0.003). The EF of healthy was higher than severe (*p* < 0.001), severe-moderate (*p* = 0.003), and moderate-mild (*p* < 0.001), and the EF of mild patients was higher than severe patients (*p* < 0.001).

#### Hybrid parameters

The SV was lower for healthy participants, but no statistically significant differences were found. The EV of healthy participants was higher than severe (*p* < 0.001), severe-moderate (*p *= 0.003), and moderate-mild patients (*p* < 0.001). The WV of healthy participants was higher than severe (*p* < 0.001), severe-moderate (*p* < 0.001), and moderate-mild patients (*p* < 0.001), and the VW of mild patients was higher than severe patients (*p* < 0.001). The NJ was lower for healthy subjects since the smoothness of movement was higher. In particular, the NJ of severe-moderate patients was higher than healthy (*p* < 0.001).

#### Compensatory parameters

Head displacements were higher for all the patients with respect to healthy participants. The HF of all groups of patients was higher than healthy (severe *p* = 0.001; severe-moderate *p* = 0.002; moderate-mild *p* = 0.004; mild *p* = 0.001); the HV of all groups of patients was higher than healthy (severe *p* < 0.001; severe-moderate *p* < 0.001; moderate-mild *p* < 0.001; mild *p* = 0.003), and the HV of severe-moderate patients was higher than mild patients (*p* = 0.004). The TT showed no significant differences among groups. The TF of severe (*p* = 0.003), severe-moderate (*p* = 0.002), and moderate-mild (*p* = 0.004) patients was higher than healthy. The TL of healthy was lower than severe (*p* < 0.001) and moderate-mild patients (*p* = 0.001), and the TL of severe patients was greater than moderate-mild (*p* < 0.001).

**Fig. 2:**
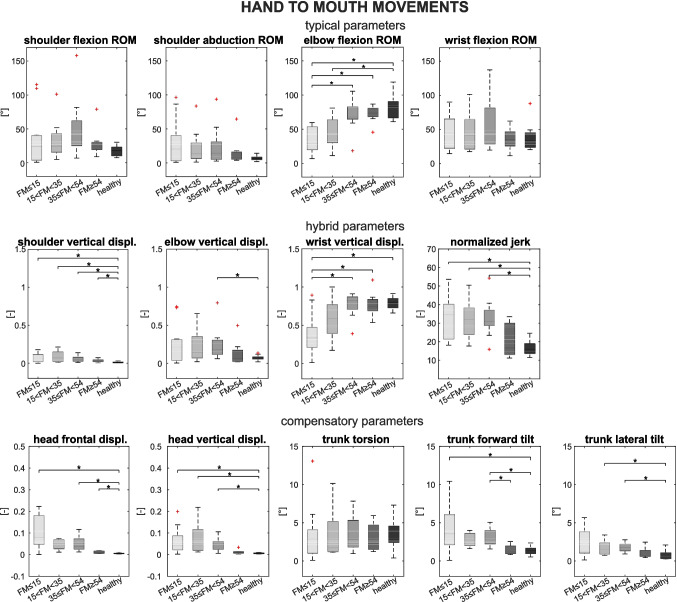
Parameters of the assessment for the HTM movement: in the first row, the distributions of typical parameters (SF, SA, EF, WF) are reported. In the second row, the distributions of hybrid parameters (SV, EV, WV, NJ) are reported. In the last row, the distributions of compensatory parameters (HF, HV, TT, TF, TL) are reported. Patient subgroups are shown in gray shades, and healthy participants are shown in black

A summary of the results for HTM movements is shown in Fig. 2 and statistical comparisons are summarized in Table 2.

**Table 2:** Mean inter-subject values of the parameters of the assessment for the HTM movement. only significant *p*-values resulting from tests performed for all pairs between groups are reported

	FM≤15	15 < FM < 35	35≤FM < 54	FM≥54	Healthy	*p*
SF [°]	33.7 (41.7)	33.9 (30.7)	48.3 (46.5)	29.3 (21.3)	17.5 (7.4)	
SA [°]	30.6 (33.4)	23.0 (27.5)	25.1 (29.9)	16.1 (20.0)	6.72 (2.9)	
EF [°]	34.8 (18.3)	46.8 (23.2)	69.4 (23.9)	72.2 (12.7)	82.4 (15.9)	FM≤15 vs. h: *p* < 0.001 15 < FM < 35 vs. h: *p* < 0.001 35≤FM < 54 vs. h: *p* = 0.002 15 < FM < 35 vs. FM≥54: *p* < 0.001
WF [°]	47.1 (24.7)	46.5 (31.1)	58.3 (39.7)	36.1 (16.3)	36.8 (17.1)	
SV [-]	0.07 (0.06)	0.09 (0.08)	0.06 (0.04)	0.03 (0.02)	0.01 (0.01)	FM≤15 vs. h: *p* = 0.001 15 < FM < 35 vs. h: p < 0.001 35≤FM < 54 vs. h: *p* < 0.001 FM≥54 vs. h: *p* = 0.003
EV [-]	0.25 (0.26)	0.23 (0.21)	0.25 (0.22)	0.13 (0.16)	0.07 (0.03)	35≤FM < 54 vs. h: *p* = 0.003
WV [-]	0.39 (0.28)	0.59 (0.27)	0.76 (0.16)	0.77 (0.16)	0.79 (0.07)	FM≤15 vs. h: *p* < 0.001 FM≤15vs. FM≥54: *p* = 0.003 FM≤15vs. 35≤FM < 54: *p* = 0.002
NJ [-]	32.5 (11.5)	32.1 (10.8)	33.0 (10.7)	20.5 (9.19)	16.6 (3.9)	FM≤15 vs. h: *p* < 0.001 15 < FM < 35 vs. h: *p* < 0.001 35≤FM < 54 vs. h: *p* < 0.001
HF [-]	0.10 (0.08)	0.11 (0.19)	0.05 (0.04)	0.01 (0.006)	0.005 (0.003)	FM≤15 vs. h: *p* < 0.001 35≤FM < 54 vs. h: *p* < 0.001 FM≥54 vs. h: *p* = 0.001
HV [-]	0.06 (0.06)	0.07 (0.08)	0.05 (0.03)	0.01 (0.01)	0.005 (0.003)	FM≤15 vs. h: *p* < 0.001 15 < FM < 35 vs. h: *p* < 0.001 35≤FM < 54 vs. h: *p* < 0.001
TT [°]	3.49 (3.65)	3.43 (3.23)	3.55 (2.42)	3.03 (1.88)	3.44 (1.90)	
TF [°]	4.12 (2.91)	4.37 (5.07)	3.06 (1.12)	1.44 (0.66)	1.35 (0.46)	FM≤15 vs. h: *p* < 0.001 35≤FM < 54 vs. h: *p* < 0.001 35≤FM < 54 vs. FM≥54 *p* = 0.003
TL [°]	2.14 (1.87)	1.72 (1.01)	1.79 (0.61)	1.04 (0.68)	0.75 (0.49)	FM≤15 vs. h: *p* = 0.003 35≤FM < 54 vs. h: *p* < 0.001

#### Typical parameters

The EF of severe was lower than healthy (*p* < 0.001), mild (*p* < 0.001), and moderate-mild patients (*p* = 0.002); the EF of severe-moderate was lower than healthy (*p* < 0.001).

#### Hybrid parameters

The SV of healthy participants was lower than all groups of patients (severe *p* = 0.001; severe-moderate *p* < 0.001; moderate-mild *p* < 0.001; mild *p* = 0.003). The EV of healthy was lower than moderate-mild patients (*p* = 0.003); the WV of severe patients was lower than healthy (*p* < 0.001), mild (*p* = 0.003), and moderate-mild patients (*p* = 0.002). The NJ of healthy subjects was lower than severe (*p* < 0.001), severe-moderate (*p* < 0.001), and moderate-mild patients (*p* < 0.001).

#### Compensatory parameters

The HF of healthy was lower than severe (*p* < 0.001), moderate-mild (*p* < 0.001), and mild patients (*p* = 0.001). The HV of healthy was lower than severe (*p* < 0.001), severe-moderate (*p* < 0.001), and moderate-mild patients (*p* < 0.001). The TT showed no significant differences among groups. The TF of healthy was lower than severe (*p* < 0.001) and moderate-mild patients (*p* < 0.001), and the TF of mild patients was lower than moderate-mild patients (*p* = 0.003). The TL of healthy was lower than severe-moderate (*p* = 0.003) and moderate-mild patients (*p* < 0.001).

## Discussion

### Principal results

In this work, the feasibility of portable, low-cost sensors for assessing motion tracking was investigated by applying a kinematic protocol based on functional movements, already used in clinics. This protocol allowed motor assessment of adults and post-stroke patients with the Kinect V2. This work confirmed previously reported findings that showed the feasibility of such approaches in the rehabilitation [35] and the industrial fields [36] for human monitoring and evaluation. We found that in both the RCH and HTM, our assessment could discriminate between various levels of disability, while only in some specific cases mild patients could be differentiated from healthy people. Interestingly, the differences were spotted mainly in head compensatory movements, highlighting the relevance of compensatory assessment in defining people’s motor capability. Severe patients showed reduced ROM in typical parameters with respect to mild patients and healthy participants in RCH. In HTM movements, instead, severe patients showed a reduced elbow ROM compared to healthy, mild, and moderate-mild individuals. However, severe-moderate and severe patients could not be discriminated using only typical parameters, as well as mild patients and healthy participants. In the hybrid parameters, severe, severe-moderate, and moderate-mild patients showed higher shoulder and lower wrist displacements in RCH; in HTM, instead, all patients showed larger shoulder displacements. Moreover, all patients, except mild, showed a reduced smoothness of movement with respect to healthy participants in HTM. Since these parameters can also be related to compensatory strategies, this result indicated that patients with stronger motor deficits use joint displacement as compensation. The compensatory parameters, especially the head displacement in both movements, allowed us to discriminate all groups of patients with respect to healthy participants, indicating that compensatory strategies are adopted by patients regardless of the level of motor impairment.

### Comparison with prior work

Kinematic analysis of patients was already studied in the literature with marker-based tracking systems, showing that post-stroke patients with moderate motor impairment have a reduced ROM at both shoulder and elbow joints with respect to healthy controls, which can be discriminated with kinematic assessment [37, 38]. Some studies already investigated the biomechanical assessment of the upper limb with low-cost portable devices. In Scano et al. [39], the performance of the patients’ ipsilateral limb was evaluated in reaching movements, and shoulder and elbow ROMs were lower than those of healthy participants, showing that a biomechanical assessment is able to capture slight angle differences, such as those found in the ipsilesional limb of post-stroke patients. Moreover, in that study, the investigators spotted no differences in trunk torsion as it was found in our study. Another study that employed Kinect V2 for motor assessment showed similar results, finding that in reaching movements, post-stroke patients with different levels of disability flexed the shoulder less and extended the elbow less, resulting in a lower ROM with respect to healthy controls [21]. Kinematic compensatory strategies, instead, were less investigated in the literature. Indeed, trunk movements were mainly assessed with marker-based systems, showing that post-stroke patients had abnormal movements and displacements of the trunk during reaching [37, 40]. Trunk displacement was also observed when reaching movements were performed with the ipsilateral upper limb [41]. Only in [21, 30], the trunk compensatory movements were investigated with markerless devices, finding that the trunk was used as compensation in reaching tasks in post-stroke patients with mild to severe impairment.

Our findings suggest that low-cost tools can discriminate different levels of impairments, as already suggested by previous research, and identify features of patients’ movement, such as the compensatory strategies adopted. Assessment of more compensatory parameters was added in this study with respect to previous works, such as head displacements. Markerless systems introduce quantitative assessments into clinics and in the home environment, increasing the possibility of executing tests for the evaluation of motor functionality in a fast and easy-to-use setup. Kinematic assessment gives a quantitative measure of motor impairment and can discriminate compensation strategies from motor recovery. In fact, assessments based on clinical scales can only be insufficiently sensitive to capture the quality of sensorimotor performance, preventing to clearly distinguish motor recovery from compensation [42, 43], which is essential to understand neurological mechanisms of sensorimotor recovery post-stroke [44]. In this way, fast protocols, without the need of attaching sensors to the patients, may improve patients’ comfort and reduce their stress that can affect the assessment [45]. Moreover, these sensors can reduce the costs related to the purchase of marker-based sensors, and there is no need for specific staff to be trained. This protocol can be integrated into the clinical assessment in order to provide a complete evaluation [46]. Then, Kinect cameras can also be used during telerehabilitation to improve the accessibility and efficiency of the rehabilitation and to guarantee the continuity of care [16]. The main drawback of using Kinect sensors is that the accuracy of distal joint reconstruction may be low and, therefore, they cannot be feasible for assessing fine movements, such as hand and finger movements [24, 47].

### Limitations

This work has some limitations. The protocol included only kinematic-derived parameters. However, compensatory strategies can also be evaluated with biomechanical and muscular assessments [11] and markerless tracking sensors demonstrated to be used for complete biomechanical assessments [36]. Future work will include kinetic measures to evaluate also the biomechanics of compensatory strategies and to increase the number of assessment parameters so that patients’ biomechanical characterization may benefit from a more refined assessment. Moreover, thirty-six patients were included in this study; involving more participants would increase the statistical power of our results. Two types of movements only were investigated, and future work could extend the applicability of this protocol to a variety of daily life movements. Furthermore, in this study, no assessment was done on the ipsilateral upper limb; including the less affected limb may give further insights into the motor strategies used by patients, since the ipsilateral limb may also show motor deficit [39]. The ipsilateral side may be involved in compensatory strategies, and this mechanism may also affect the neural re-organization and adaptation after stroke [6]. Therefore, future studies will also consider the ipsilateral upper limb in the analysis. Finally, other new technologies are emerging for kinematic markerless tracking, such as head-mounted displays (HMDs) in virtual reality (VR) systems. These systems have been demonstrated to be feasible for both assessment and rehabilitation [48]. Indeed, their accuracy was tested and compared to marker-based tracking systems in shoulder rehabilitation [49] and in hand movements in post-stroke patients [50, 51]. The HMDs allow free movement and an immersive scenario, allowing the execution of highly realistic and task-oriented movements. HMDs have been demonstrated to be well-tolerated and promising for increasing motor function in adult chronic stroke survivors, with benefits in subjects’ arm use and independence [52]. This new technology can be a valuable instrument for applying a biomechanical assessment in future studies for the evaluation of compensatory strategies, since it allows realistic movement in an immersive scenario, and future evaluations may compare the use of HMDs with the Kinect-based assessment.

## Conclusions

In this paper, we provide evidence that low-cost, instrumental assessments of post-stroke patients can be quantified effectively, and compensation strategies correlate with clinical scales. A kinematic protocol was used for assessing four groups of patients with different levels of motor impairment. Kinematics measures discriminated severe and moderate patients from healthy controls. Compensatory parameters, instead, can also distinguish mild patients from healthy participants. Markerless systems demonstrated their feasibility to be used for clinical assessment, introducing quantitative measures into clinics and in the home environment, increasing the possibility of executing tests for the evaluation of motor functionality in a fast and easy-to-use setup.

Moreover, the possibility to study, using clear and reliable outcome measures, the compensatory strategies and biomechanics of the upper limb function of post-stroke individuals may permit to better characterize the patient and guide rehabilitation, tailoring technologies, robotics, and physiotherapy.

## Data Availability

The data presented in this study are available on request from the corresponding authors.

## Abbreviations

*EF*: Elbow flexion
*EV*: Elbow vertical displacement
*FM*: Fugl-Meyer Assessment
*HDM*: Head-mounted display
*HF*: Head frontal displacement
*HTM*: Hand to mouth movement
*HV*: Head vertical displacement
*NJ*: Normalized jerk
*RCH*: Reaching movement
*SA*: Shoulder abduction
*SF*: Shoulder flexion
*SV*: Shoulder vertical displacement
*TF*: Trunk forward/backward tilt
*TL*: Trunk lateral tilt
*TT*: Trunk torsion
*VR*: Virtual reality
*WF*: Wrist flexion
*WMFT*: Wolf Motor Function Test
*WV*: Wrist vertical displacement
